# Glucose-activatable insulin delivery with charge-conversional polyelectrolyte multilayers for diabetes care

**DOI:** 10.3389/fbioe.2022.996763

**Published:** 2022-09-29

**Authors:** Yanguang Yang, Xiangqian Wang, Xiaopeng Yuan, Qiwei Zhu, Shusen Chen, Donglin Xia

**Affiliations:** ^1^ Department of Radiotherapy, Nantong Tumor Hospital, Tumor Hospital Affiliated to Nantong University, Nantong, China; ^2^ School of Public Health, Nantong University, Nantong, China

**Keywords:** glucose-activity, diabetes, insulin, charge shifting, layer-by-layer

## Abstract

One of the most effective treatments for diabetes is to design a glucose-regulated insulin (INS) delivery system that could adjust the INS release time and rate to reduce diabetes-related complications. Here, mixed multiple layer-by-layer (mmLbL)-INS microspheres were developed for glucose-mediated INS release and an enhanced hypoglycemic effect for diabetes care. To achieve ultrafast glucose-activated INS release, glucose oxidase (GOx) was assembled with a positively charged polymer and modified on INS LbL. The mmLbL-INS microspheres were constructed with one, two, and four layers of the polyelectrolyte LbL assembly at a ratio of 1:1:1. Under hyperglycemia, GOx converts a change in the hyperglycemic environment to a pH stimulus, thus providing sufficient hydrogen ion. The accumulated hydrogen ion starts LbL charge shifting, and anionic polymers are converted to cationic polymers through hydrolytic cleavage of amine-functionalized side chains. The results of *in vitro* INS release suggested that glucose can modulate the mmLbL-INS microspheres in a pulsatile profile. *In vivo* studies validated that this formulation enhanced the hypoglycemic effect in STZ-induced diabetic rats within 2 h of subcutaneous administration and facilitated stabilization of blood glucose levels for up to 2 days. This glucose-activatable LbL microsphere system could serve as a powerful tool for constructing a precisely controlled release system.

## Introduction

In diabetes care, maintaining stable blood glucose levels (BGLs) in normoglycemia is a must because hyperglycemia would induce secondary complications, such as vascular, renal, or neural damage (Kang, 2018; Kaura Parbhakar et al., 2020; Shah and Wu, 2022). Most patients with type 2 diabetes need direct intravenous exogenous insulin (INS), and therefore, they are administered multiple injections of INS with careful dosing, which ultimately results in poor compliance (Ling and Chen, 2013). Various INS delivery systems are sensitive to different external stimuli, such as pH (Akhmedov et al., 2010; Yoshida et al., 2012; Dmitriev et al., 2019), temperature (Huynh et al., 2008; Killeen et al., 2020), or light (Solimena and Speier, 2010; Sarode et al., 2016; Yu et al., 2022), were investigated. However, most INS delivery systems are associated with a lag time of more than 2 h in hypoglycemia. Thus, precise control of the amount and rate of INS released into the blood in order to maintain the BGL within the narrow concentration window required to avoid hyperglycemia and hypoglycemia is challenging.

The layer-by-layer (LbL) assembly method relies on solid exfoliation to produce colloids of sheets and can control drug release under particular external stimulus (Baba et al., 2010; Huang et al., 2013; Zheng et al., 2014; Jiang and Kobayashi, 2017; Zhang et al., 2021). LbL drug administration has showed some advantages in the precise control of size, or membrane thickness at the nanoscale level (Balabushevich et al., 2003; Xing et al., 2007). Although great success has been obtained with these LbL-modified INS therapies in controlled release, they exhibited a slow rate of INS release, which restricted timely BGL control, probably because of mass transport limitation (Ravaine et al., 2008). The rate of INS release needs to be improved to enhance the effects of glycemic control.

Blood glucose-activated drug release systems allow rapid and precisely controlled responses to changes in BGLs, thereby ensuring good glycemic control (Xia et al., 2018). In the past few decades, many studies have focused on developing glucose-responsive systems capable of continuously delivering accurate levels of INS in response to the BGL because it is immediate and effective (Chen et al., 2011; Paez et al., 2013; Li et al., 2015; Llanos et al., 2015; Kompala and Neinstein, 2022). To achieve the desired glucose-activated INS release, glucose oxidase (GOx) and a positively charged polymer were assembled with INS to form multilayer films (Chen et al., 2012). These multilayer films showed a linear release and exhibited the desired on–off sensitivity in response to stepwise glucose challenge. Gu et al. also reported that a useful glucose-mediated INS delivery platform consisting of GOx exhibited good results (Gu et al., 2013). GOx plays a major role in the construction of glucose-activated controlled INS release devices (Chu et al., 2012).

**FIGURE 1:**
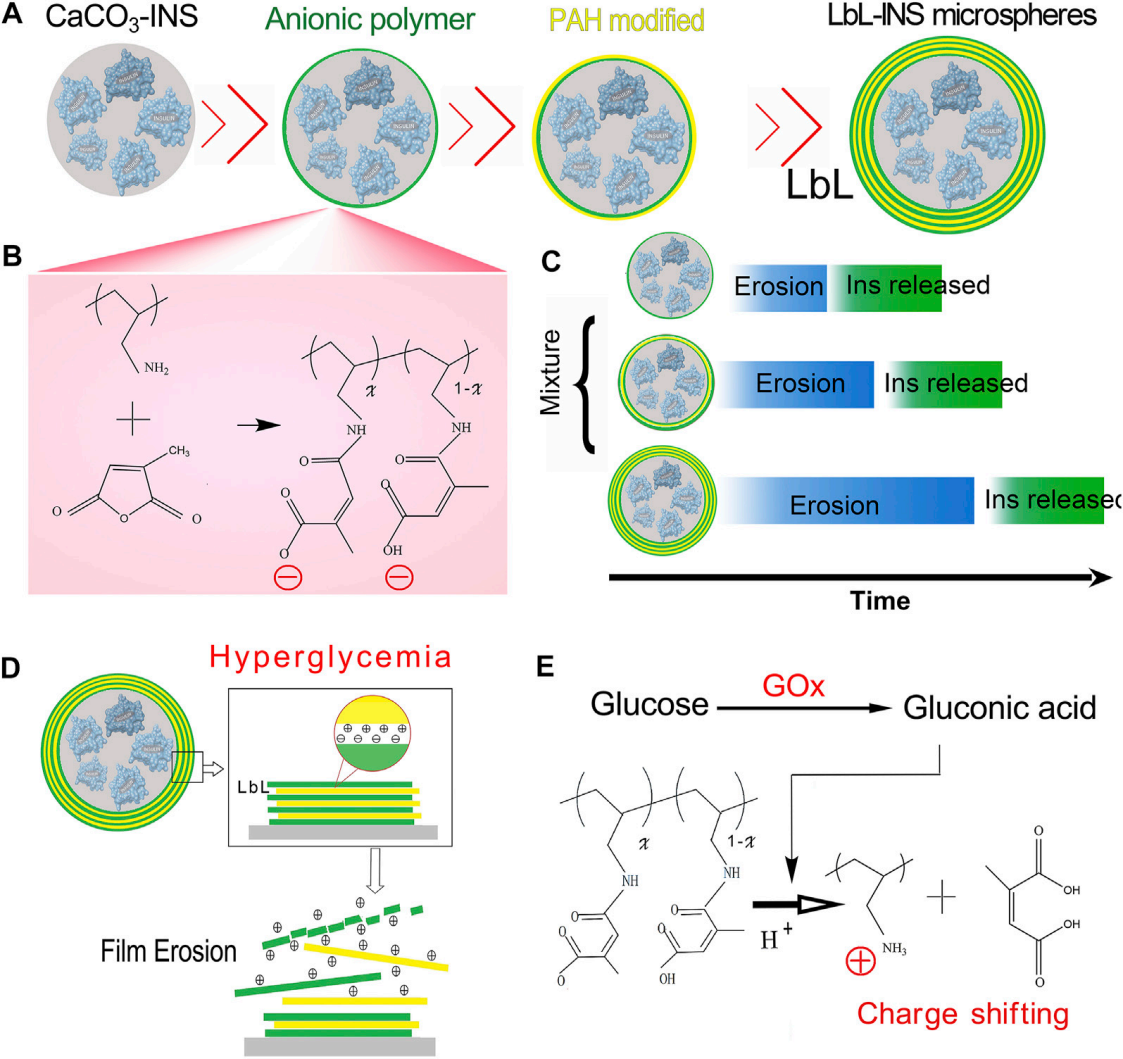
Scheme of the mixed multiple layer-by-layer insulin (mmLbL-INS) microcapsules. **(A)** Synthesis of the multiple LbL-INS microcapsules. The anionic polymer was regarded stable in physiologically relevant environments and hydrolysis of the charge-shifting anionic polymer under acidic conditions yields a cationic polymer. Based on the LbL assembly of multilayered polyelectrolyte films, with repeat assembly sequences polycation PAH and anionic polymer, we fabricated LbL-INS microspheres with different layers. **(B)** Synthesis of degradable anionic polymers. **(C)** Schematic of mmLbL-INS microcapsules controlling the INS release time. **(D)** In hyperglycemia, glucose-activated changes in the net charge of the polymer led to changes in the nature of ionic interactions in the multilayers and promoted film disruption. **(E)** Hydrolysis of the citraconic amide side chains of the charge-shifting anionic polymer under acidic conditions.

An anionic polymer can rapidly convert back to a cationic polymer in acidic environments within seconds. In this study, mixed multiple layer-by-layer (mmLbL)-INS microspheres were developed for glucose-mediated INS release and an enhanced hypoglycemic effect for diabetes care (Figure 1). The mmLbL-INS microspheres were constructed with one, two, and four layers of the polyelectrolyte LbL assembly at a ratio of 1:1:1. We anticipate that the resulting mmLbL-INS microspheres can be used for glucose-regulated INS delivery to enhance the hypoglycemic effect and maintain blood glucose homeostasis to reduce complications in diabetic patients.

## Materials and methods

### Materials and animals

Poly (allylamine hydrochloride) (PAH, MW ≈ 60,000) was purchased from Alfa Aesar Organics (Ward Hill, MA). Recombinant cow INS (potency: 27 U/mg) was obtained from Bomei Biotechnology (Hefei, China). Citraconic anhydride and poly (styrene sulfonate) (PSS) were purchased from J&K Scientific (Beijing, China). GOx was obtained by Tokyo chemical industry (Tokyo, Japan). Streptozocin (STZ) was purchased from Sigma-Aldrich, (St. Louis, MO, United States). The dialysis bag (molecular cutoff, 3500 Da) was purchased from Sangon Biotech (Shanghai, China). All other reagents were used without further purification unless noted.

The Sprague–Dawley (SD) rats (275–300 g) were provided by the Experimental Animal Center of Nantong University. All animal experiments were performed in compliance with all relevant ethical regulations and institutional guidelines provided by the Division of Comparative Medicine at Nantong University.

### Synthesis of charge-shifting anionic polymers

The synthetic route used for degradable anionic polymers is shown in Figure 1B, which was reported in a previous paper (Liu et al., 2008). Briefly, 100 mg PAH was dissolved in 1.0 M NaOH (4 ml) and stirred until completely dissolved. Then, 400 μL citraconic anhydride was added dropwise to PAH solution, and aqueous NaOH was added as necessary to maintain the pH above 8. Finally, the resulting reaction mixture was placed in dialysis bag (MWCO = 3500) and no reacting parts were removed by dialysis with water (pH > 7.4) for 24 h.

### Preparation of premixed LbL-INS microcapsules

The cores of the microcapsules were fabricated according to early reported (Bosio et al., 2014). In brief, 0.35 g Na_2_CO_3_, 0.02 g PSS and certain amounts of INS was added to 10 ml distilled water, then 10 ml CaCl_2_ solution (0.33 M) was added and vigorous stirred for 10 s. The obtained microparticles were washed for three times and were separated by centrifugation.

Multilayered films were fabricated on the INS microcapsules using an alternate dipping procedure according to the following general protocol: microcapsules are submerged in a solution of anionic polymers for 5 min, and washed with water (pH > 7.4), anionic polymers modified microcapsules were submerged in a solution with excess of PAH for 5 min, and washed. The following two, three and four bilayers were fabricated in the same way by alternating adsorptions of anionic polymers and PAH as shown in Figure 1A. The premixed LbL-INS microcapsules with 1, 2, four layers at the rate of 1:1:1 was used in this study.

### Scanning electron microscopy and zeta potentials

To investigate morphological changes in mmLbL-INS microspheres after the LbL process, the morphologies of CaCO_3_-INS and four-bilayered microspheres were selected for characterization. The surface morphology of mmLbL-INS microspheres corroded in different pHs was also investigated through scanning electron microscopy (SEM) (JSM-6700F, JEOL, Japan) on conductive adhesive tapes (Briones et al., 2010).

LBL deposition of anionic polymers/PAH on the particles and the charge conversion in different pH were qualitatively monitored by measuring the zeta potentials of the coated particles by using Zetasizer Nano ZS (Malvern, Worcestershire, United Kingdom).

### Drug loading content

According to our previous study, a HPLC detection method was established to determine the INS concentration (Xia et al., 2018). HPLC was performed using a LC-2030 series (Shimadzu Corporation, Japan).

Then, 1, 3, 5, and 10 mg of INS (WT) was dissolved in CaCl_2_ solution in advance, followed by the preparation of the four-bilayered LbL-INS microspheres. INS was encapsulated in the LbL-INS microspheres using a hypotonic dialysis method. The LbL-INS microspheres (3 mg/ml) were dispersed in dilute hydrochloric acid, and a clear solution was used for measurement with HPLC. The loading content can be calculated as follows.

$$Drug\;loading\;content\;\left(\%\right)=\frac{Total\;mass\;of\;INS\;in\;the\;microspheres}{Total\;mass\;of\;the\;microspheres}\times 100\%$$

### Release rate tests

After being suspended in 100 mg/dl (equivalent of 5.6 mM) d-glucose for 10 days, the mmLbL-INS microspheres were incubated in PBS with 400 mg/dl (equivalent of 22.2 mM) glucose *in vitro*. The INS released from the mmLbL-INS microspheres was collected at predetermined time intervals and detected through HPLC. Then, the release rates were calculated.

### *In vitro* testing of release behavior of mmLbL-INS microspheres and fluorescence microscopy analysis

To verify the effect of “charge-conversional”, LbL-INS microspheres were resuspended in PBS containing different glucose concentrations at 37°C to study the INS release behavior. At predetermined times, the amounts of INS released into the supernatant was determined through HPLC, and morphological changes were investigated through SEM.

INS-FITC (green) and rhodamine B (red)-modified GOx were packed in the LbL-INS microspheres and resuspended in PBS containing different glucose concentrations. Fluorescence microscopy was performed using a Leica DM 2500 microscope (Wetzlar, Germany) at the same laser power.

### *In vivo* oral glucose tolerance test in STZ diabetic rats

STZ diabetic rats were prepared by treated with STZ (50 mg/kg) into male SD rats. The rats were considered diabetic if their plasma glucose levels were ≥10 mM (Shao et al., 2021).

The diabetic rats were separated into three groups (n = 8): Control group: 1 ml PBS was subcutaneously injected; CaCO_3_-INS group: 1 ml of 0.035 mg/ml CaCO_3_-INS microspheres (the diabetic rats treated with >0.035 mg/ml CaCO_3_-INS microspheres died within 15 min because of hypoglycemia. So, in the following studies, 0.035 mg/ml of CaCO_3_-INS microspheres were used in *in vivo* studies); and mmLbL-INS microspheres group: 1 ml of mmLbL-INS microspheres was subcutaneously injected. The oral glucose tolerance test (OGTT) was performed as described by Kim (Kim et al., 2016). In brief, diabetic rats were orally administered glucose (2.0 g/kg b.w), blood was collected from the tail vein to measure blood glucose and INS concentrations. The plasma glucose was measured using a hand-held glucometer at regular intervals (0, 15, 30, 60, 120 min). Blood was collected from the tail vein 0, 15, 30, 45, 60, 75, 90, 105 and 120 min after glucose administration. The blood INS was then quantified via HPLC as previous report (Xia et al., 2018).

### Long-term effect of mmLbL-INS microspheres

The diabetic rats were subcutaneously injected with PBS (blank), CaCO_3_-INS microspheres (0.035 mg/ml), and mmLbL-INS microspheres (1 mg/ml) into the back. Each diabetic rat was individually housed in metabolic cages and fed with maintenance diets (Xietong Engineering Co., Ltd, referring to the national standard GB 14924.1-2001) 3 times daily. 0.5 h after every feeding, leftover food was removed, and feed intakes were measured. After feeding for 2 h, BGLs were measured.

### Hematological indices

Hematological parameters such as hematocrit (HCT), released hemoglobin (HGB), mean corpuscular volume (MCV), mean corpuscular hemoglobin (MCH), and mean corpuscular hemoglobin concentration (MCHC) were determined using an HEMAVET 950FS Hematology System analyzer after the administration of LbL-INS microspheres for 24 h.

### Blood biochemistry index

After administering mmLbL-INS microspheres for 2 days, the levels of liver function markers, including alkaline phosphatase (ALP), alanine aminotransferase (ALT), and aspartate aminotransferase (AST), the kidney function marker urea nitrogen (BUN), as well as creatinine (Cr), were determined using an automated biochemical analyzer (Trilogy, France).

### Statistical analysis

All values were averaged and expressed as the mean ± standard error of the mean. Analysis of variance was performed, with dialysis time, initial INS concentration, and volume ratio as three independent variables. *p* values ≤0.05 were considered significant.

## Results

### Preparation of LbL-INS microspheres

**FIGURE 2:**
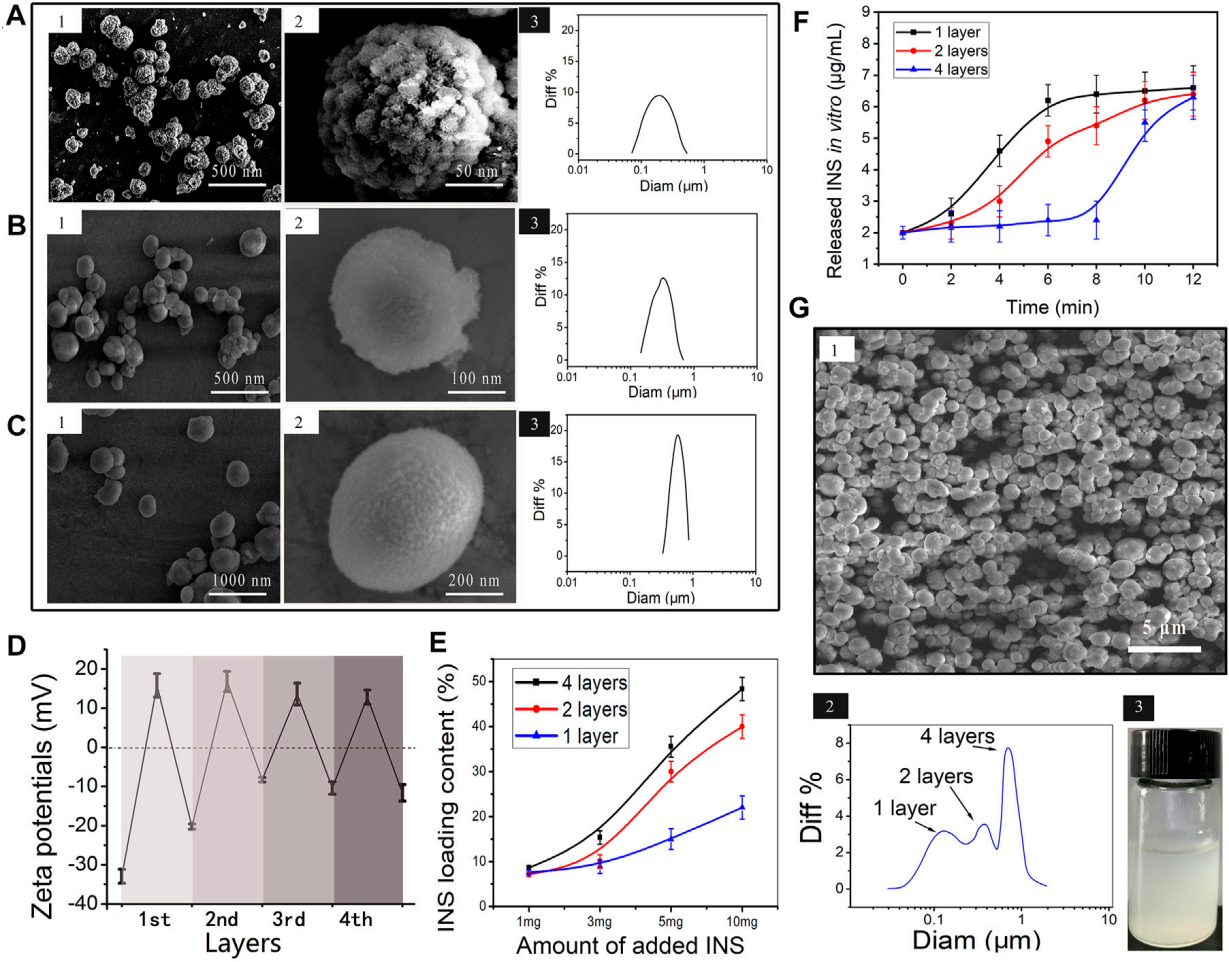
Characterization of the mmLbL-INS microcapsules. **(A–C)** SEM images and DLS results of LbL-INS microcapsules with **(A)** one layer, **(B)** two layers, and **(C)** four layers. (1) SEM images, (2) high-magnification SEM image, (3) dynamic light scattering (DLS) results. **(D)** Zeta potentials of multiple LbL-INS microcapsules with different layers. **(E)** Relationship between the initial INS concentration and INS loading content in the LbL-INS microcapsules. **(F)** INS release behavior of multiple LbL-INS microcapsules in pH 6.5 solution. **(G)** SEM images, DLS results, and the image of mmLbL-INS microcapsules.

In this study, the microspheres were prepared by the LBL assembly of multilayered polyelectrolyte films, with repeat assembly sequences polycation PAH and anionic polymer, as shown in Figure 1B. Morphological changes in the LbL-INS microcapsule with different polyelectrolyte films were monitored through SEM (Figures 2A–C). CaCO_3_-INS microparticles with the one-layer polyelectrolyte film showed clearly visible wrinkles and inward depression on the surface (Figure 2A). The surface of the LbL-INS microspheres became smooth and glossy as the layers increased (Figures 2B, C). The average diameter of the LbL-INS microspheres, through dynamic light scattering (DLS), increased slightly with an increase in the number of layers (Supplementary Figure S1).

The LbL assembly process was also monitored through zeta-potential measurements (Figure 2D). The uncoated CaCO_3_ microparticles produced a negative zeta-potential (−32 mV). The average zeta-potential value for the first layer increased to 14 mV, corresponding to the adsorption of the polycation PAH, that for the second layer (−20 mV) corresponded to the adsorption of the anionic polymer, that for the third layer (15 mV) corresponded to another layer of PAH, and that for the fourth layer (−10 mV) corresponded to a new layer of anionic polymer. Alternating zeta-potentials were obtained with the subsequent deposition of PAH and polyanions, suggesting the gradual adsorption of the PAH/anionic polymer layers on the CaCO_3_-INS microparticles.

Not unexpectedly, the loading content was significantly positively correlated with the initial INS concentration, not only in one-layer LbL-INS microspheres but also in two- and four-layer LbL-INS microspheres (Figure 2E). Increasing the layers of microspheres resulted in high INS loading content. Combining the results in Figures 1A–C, we speculated that the polycation PAH and anionic polymer modified on the CaCO_3_-microparticles could prevent INS leakage, leading to an increase in INS loading content. In consideration of the loading efficiency and cost-savings, in the following experiment, an initial INS concentration of 5 mg/ml was adopted to achieve the acceptable encapsulation concentration in mmLbL-INS microspheres.

The hyperglycemia response time of LbL-INS microspheres with different bilayers was studied (Figure 2F). The one-layer LbL-INS microspheres took approximately 4.1 ± 0.4 min to transform to explosive release. Relatively, the multilayered polyelectrolyte films on the four-layer LbL-INS microspheres took as long as 9.3 min to remove and start INS release. The retention time of LbL-INS microspheres extended with an increase in the number of layers. The aforementioned results revealed that mmLbL-INS microspheres with different layers showed different glucose-activated response rates. According to the characteristics of INS clinical needs (Gracia-Ramos et al., 2016; Maiorino et al., 2017), premixed INS analogs (rapid-acting and long-acting analog ratio in varying proportions) represent an alternative treatment for type 2 diabetes. In this study, mmLbL-INS microspheres constructed with one, two, and four layers at a ratio of 1:1:1 and the INS concentration of 0.98 ± 0.13 mg/ml were used in the following studies. The SEM, dynamic light scattering, and images of the mixture of LbL-INS microspheres are presented in Figure 2G. The mmLbL-INS microspheres with different layers maintained their respective structure and size.

### *In vitro* testing of glucose-activated release behavior

**FIGURE 3:**
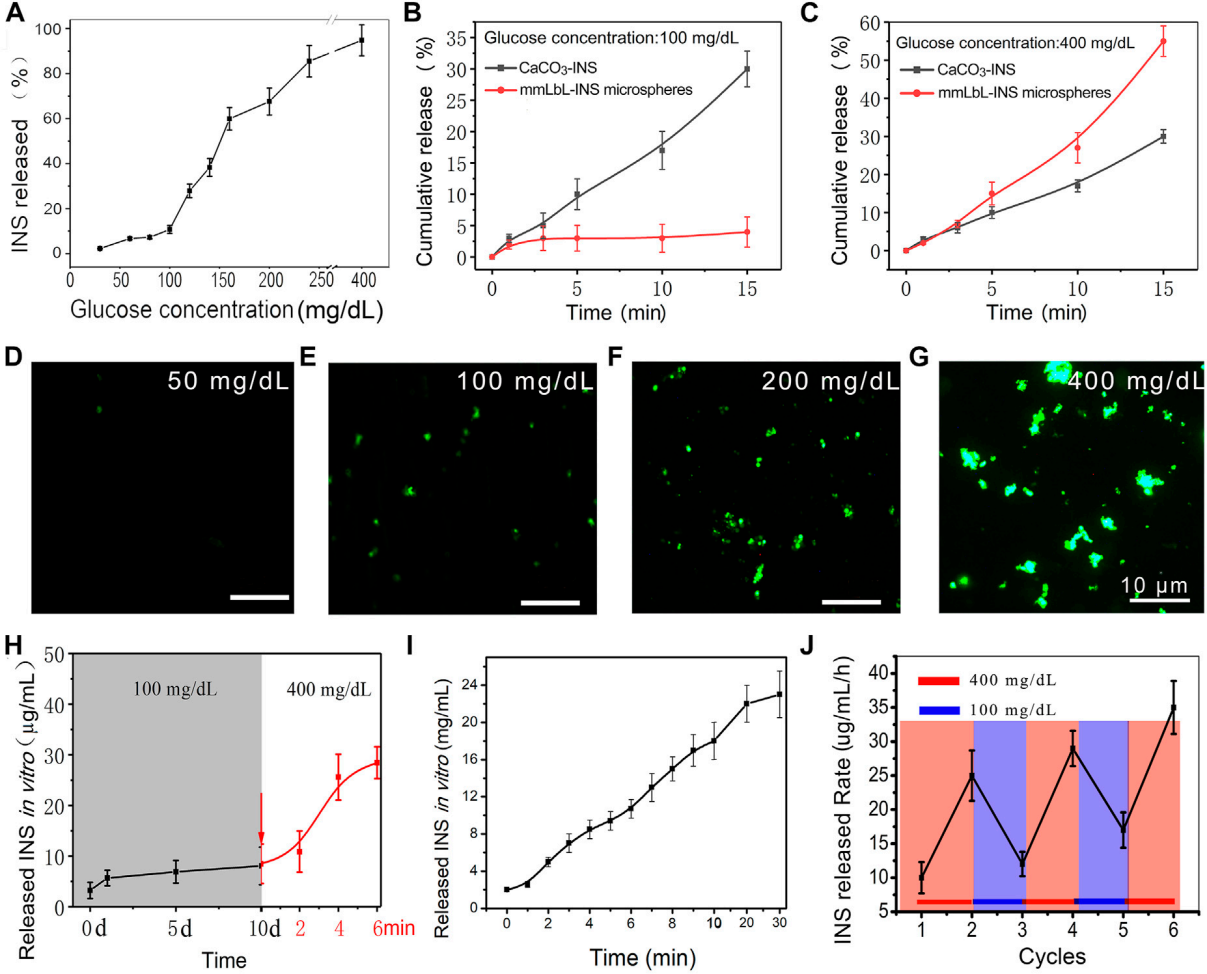
Glucose-activated INS release behavior of the mmLbL-INS microcapsules. **(A)** The INS release behavior at different glucose concentrations **(B and C)** Cumulative INS release behavior of mmLbL-INS microcapsules immersed in 100 and 400 mg/dl glucose solutions. **(D–G)** Fluorescence images of mmLbL-INS microcapsules that released INS into the medium after being immersed in different glucose concentrations (green fluorescence: FTIC-INS). **(H)** Stability analysis of the glucose activity effect. After being stored in 5 mmol/L glucose solution at 4°C for 10 days, the mmLbL-INS microcapsules released INS *in vitro* (read line) when immersed in 400 mg/dl glucose solution. **(I)** INS release behavior of the mmLbL-INS microcapsules changed over time in 400 mg/dl glucose solution. **(J)** INS release exhibited a pulsatile profile in response to different glucose concentrations. The mmLbL-INS microcapsules were immersed in normal (100 mg/dl) or hyperglycemic level (400 mg/dl) solution for several repetitions.

Next, we investigated the glucose-activated release behavior of mmLbL-INS microspheres in response to BGLs. To validate our design, mmLbL-INS microspheres were incubated in PBS (pH 7.4) at 37°C with 25–400 mg/dl glucose and INS release was monitored by HPLC. As shown in Figure 3A, increasing the glucose concentration to >100 mg/dl would greatly accelerate INS release.

In glycemia, GOx could convert glucose to glucose acid (Botezatu et al., 2021). As more and more lactic acid is produced, pH falls. Based on the principle, the mmLbL-INS microspheres were expected to show glucose-activated release behavior. To examine whether the glycemia control effect of the INS microspheres was due to sustained INS release or glucose-responsive INS release, the cumulative INS released from mmLbL-INS microspheres were determined in normal glycemia or hyperglycemia, respectively. As shown in Figures 3B,C, incubation of mmLbL-INS microspheres at the normal glucose level resulted in a slightly increased cumulative release of INS within 15 min. When the glucose concentration increased to the hyperglycemic level, >60% of INS was released by 15 min from the mmLbL-INS microspheres group. As expected, the cumulative release rate of CaCO_3_-INS microspheres increased straightly not only in hypoglycemia but also in normoglycemia.

To further assess glucose-responsive INS release, FITC-INS was used to trace the INS release behavior of mmLbL-INS microspheres. When 2 ml of the mmLbL-INS microspheres were imaged in the presence of 50 and 100 mg/dl glucose (Figures 3D,E), slight green fluorescence was found, clearly indicating that almost no INS released. When these mmLbL-INS microspheres were in hyperglycemia (Figures 3F,G), the fluorescence intensity of the supernatant enhanced remarkably (Supplementary Figure S2). The results indicated that the sensitivity of mmLbL-INS microspheres to BGLs can enhance the INS release rate.

To examine the stability of the glucose activity effect of mmLbL-INS microspheres incubated in the normoglycemic solution (100 mg/dl of glucose). After immersion for 10 days, INS concentrations in the supernatant were stable (Figure 3H). Then, the glucose concentration was immediately adjusted to 400 mg/dl, the concentration of released INS rose quickly and reached 27 μg/ml within minutes. The INS concentration in the supernatant showed a steady rise with the long-term storage of mmLbL-INS microspheres in a high sugar solution (400 mg/dl) (Figure 3I).

Furthermore, the INS release profile of mmLbL-INS microspheres exhibited a pulsatile pattern (close-loop) when the glucose concentration was cyclically varied between the normal and hyperglycemic levels for several repetitions, as shown in Figure 3J. The mmLbL-INS microspheres responded to changes in glucose levels, and the INS release rate increased to over 25 μg/mL/h in hyperglycemic state.

As more and more lactic acid was produced in hyperglycemia, because GOx in the mmLbL-INS microspheres converted glucose to glucose acid, pH fell. The charge-shifting anionic polymers on the mmLbL-INS microspheres disrupted the ultrathin polyelectrolyte multilayers in acidic media and the INS released. Contrarily, in normal glycemia, the INS released behavior stopped in the mmLbL-INS microspheres as the pH value came to normal. These results also confirmed that GOx maintains its bioactivity even after 10 days, and thus makes the delivery system highly stable.

### Release mechanism and charge-shifting effect

**FIGURE 4:**
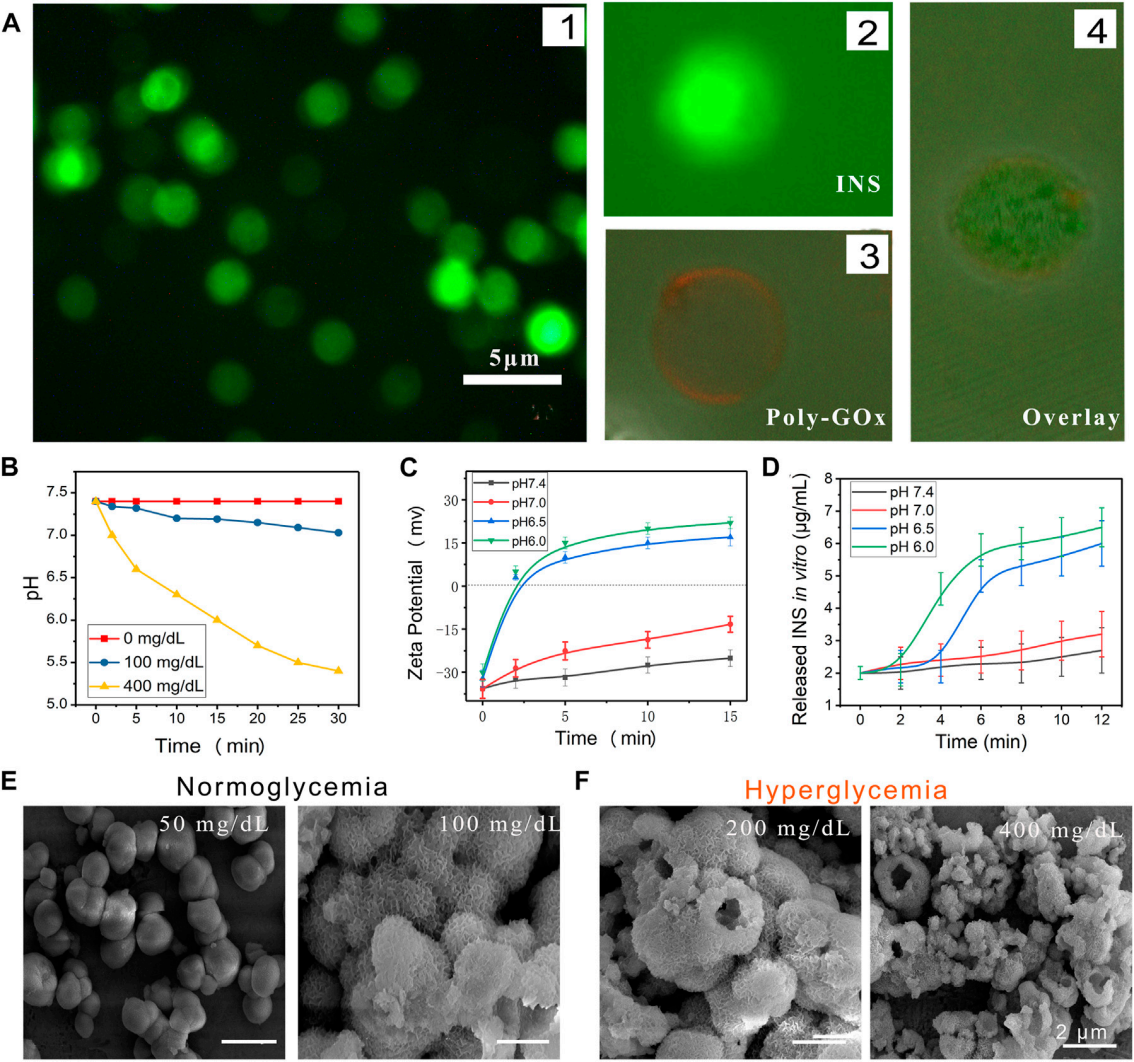
The mechanism of glucose-activated INS release behavior of mmLbL-INS microcapsules. **(A)** Fluorescence image of mmLbL-INS microcapsules. FITC-labeled INS (green) and rhodamine B (red)-modified biotin-GOx were used to fabricate mmLbL-INS microcapsules. **(B)** Relevant pH changes observed in different incubation solutions with mmLbL-INS microcapsules. **(C)** Charge conversion of anionic polymers at different pH. **(D)** INS release behavior of mmLbL-INS microcapsules in different pH solutions. **(E,F)** SEM of mmLbL-INS microcapsules immersed in different glucose concentrations.

Polyelectrolyte films and GOx, modified on the surface of LbL-INS microspheres, were expected to realize the glucose-responsive INS releasing behavior. Then, FITC was used as a tracer of INS, and visible green fluorescence observed in the solution further confirmed the absence of INS in the LbL-CaCO_3_-INS microspheres (Figure 4A). From the confocal images of LbL-INS microspheres, a red color (rhodamine B-modified GOx) could be found on the surface of the LbL-INS microspheres. In the overlay image, the green spheres were covered by red loops, which confirmed that poly-GOx was modified on the LbL-INS microspheres.

The pH of body (interstitial) fluids can be reduced by GOx, which can convert glucose to gluconic acid (Wu et al., 2011). The GOx, on the surface of LbL-INS microspheres, causing a local decrease in pH with an increase in glucose concentrations (Figure 4B). GOx catalyzes the conversion of glucose to gluconic acid as follows:

$$glucose+O_{2}+H_{2}O\to gluconic\;acid+H_{2}O_{2}$$

The charge-shifting anionic polymers can disrupt the ultrathin polyelectrolyte multilayers in acidic media. Based on this understanding, we designed a glucose-activated INS release system by using charge-shifting anionic polymers. As the normal blood sugar range is 70–100 mg/dl (Barkoudah and Weinrauch, 2015), the *in vitro* release of INS from the mmLbL-INS microspheres was evaluated in different concentrations of glucose within the solutions. To examine the glucose-responsive dissociation of mmLbL-INS microspheres, the microspheres were collected in micro-centrifuge tubes and incubated with PBS solutions with 100 (corresponding to the normal level) or 400 mg/dl (corresponding to the hyperglycemic level) glucose. In Figure 4B, the recorded pH values decreased from 7.4 to 5.4 within 30 min in 400 mg/dl glucose, confirming the enzymatic conversion of glucose to gluconic acid. By contrast, both control samples (no glucose and 100 mg/dl glucose) showed no observable changes in pH (pH > 7.0).

The polyelectrolyte multilayer was thus expected to show a charge conversion behavior in the acid environment (Figure 4C). Compared with incubation at pH 7.4 and 7.0, the zeta potential of the polyelectrolyte multilayer increased significantly when incubated at pH 6.5 and 6.0. The zeta potential became positive within 2 min, despite the potential of the polyelectrolyte multilayer slowly increasing with incubation at pH 7.4 and 7.0.

Charge-shifting of the net charge of the polymer leads to changes in the nature of ionic interactions in the multilayers and promotes film disruption and the release of cationic film components (Figure 4D). To offer a rapid glucose responsibility to these mmLbL-INS microspheres, GOx was conjugated with charge-conversional polyelectrolyte multilayers in the presence of N, N′-carbonyldiimidazole. When the BGL increased, GOx catalyzed the conversion of glucose to gluconic acid in the presence of oxygen, which provided enough hydrogen ion, leading to the low pH environment (Qiu et al., 2009). When exposed to low pH environments, hydrolysis of citraconic amide side chains of the charge-shifting anionic polymer yields a cationic polymer.

Morphological changes in the mmLbL-INS microspheres immersed during the course of glucose-activated INS release were also investigated through SEM (Figures 4E,F). The mmLbL-INS microspheres submerged in 50 mg/dl of glucose showed negligible INS release and remained intact. By contrast, the LbL-INS microspheres were found to corrode the polymer and rupture the CaCO_3_ shell when exposed to a hyperglycemic environment. The CaCO_3_-INS microparticles changed from smooth to inward depression on the surface. Their fluorescence intensity increased with the glucose concentration. Collectively, these results indicate the charge-shifting effect of the LbL-INS microspheres and the subsequent glucose-mediated and pH-dependent INS release.

### *In vivo* OGTT result

**FIGURE 5:**
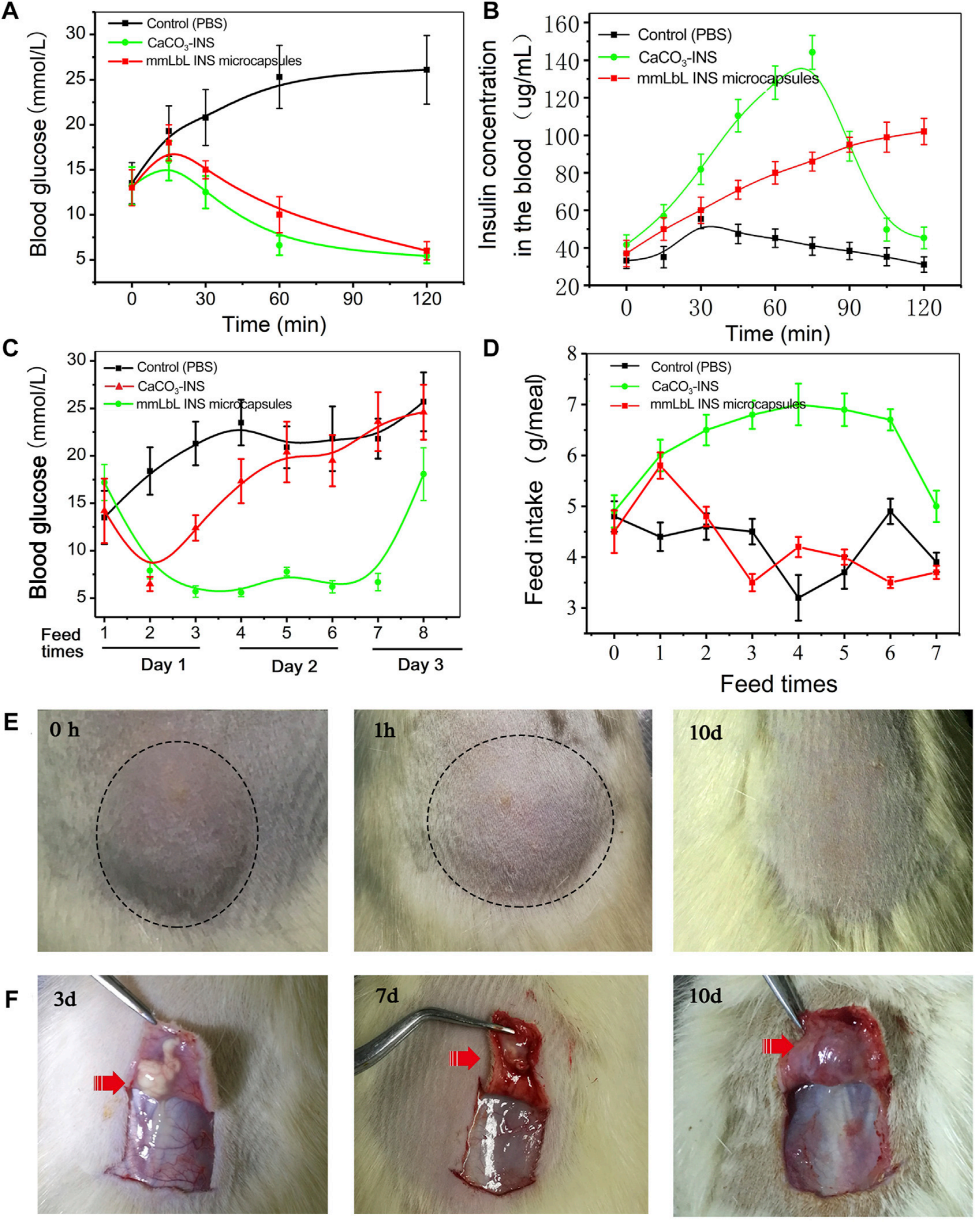
*In vivo* glycemic control tests results of mmLbL-INS microcapsules. **(A)** Change in glucose concentrations and **(B)** change in the INS concentration of the whole blood in the oral glucose tolerance test. **(C)** Long-term plasma blood glucose concentration. **(D)** Food intake in diabetic rats administered STZ. **(E)** Pictures of the injected set of mmLbL-INS microcapsules on different days. **(F)** The change of mmLbL-INS microcapsules in subcutaneous.

The OGTT was used to determine the regulation of the BGL in STZ-induced diabetic rats after treatment with 1) PBS, (b) 0.035 mg/ml CaCO_3_-INS microspheres, and (c) 1 mg/ml mmLbL-INS microspheres. Not surprisingly, the BGLs increased rapidly and were maintained over 25 mM (equivalent of 450 mg/dl) after 1 h of gastric perfusion of glucose in the PBS-treated control group (Figure 5A). As expected, the BGLs were well-controlled in the normal range of 3.9–6.1 mM within 2 h in the CaCO_3_-INS and mmLbL-INS microspheres groups, like the normal rats (Supplementary Figure S3). The blood INS levels were also measured as shown in Figure 5B. More and more INS was found as time elapsed when higher amounts of CaCO_3_-INS microspheres were applied. A peak release behavior was observed in the mmLbL-INS microspheres group, and the INS release amount decreased as the glucose concentration returned to the normal blood glucose level (Supplementary Figure S4).

### mmLbL-INS microspheres control hyperglycemia in diabetic rats

To examine the efficacy of mmLbL-INS microspheres in diabetes treatment, STZ-induced diabetic rats were subcutaneously injected with PBS solution, CaCO_3_-INS (0.035 mg/ml INS), and mmLbL-INS microspheres (1 mg/ml INS). Figure 5C showed that the mmLbL-INS microspheres could maintain normoglycemia glucose levels in diabetic rats for more than seven feed times (approximately 3 days), after which the levels gradually increased, possibly due to the depletion of the reservoir INS with time. By contrast, the control group (PBS treated) demonstrated elevated BGLs between 230 and 450 mmol/L. As the CaCO_3_-INS group released INS after dosing (could not release INS response to BGL), the BGL decreased to the normoglycemic level at first, and then increased directly with the depletion of the reservoir INS. Furthermore, the diabetic rats in the mmLbL-INS microspheres treatment group took more fodder, with no mortality (Figure 5D and Supplementary Figure S5). By contrast, diabetic rats in the control and CaCO_3_-INS groups took less fodder because of diabetic ketoacidosis. Then, in order to maintain the BGL in normoglycemia as normal rats, the diabetes should administrate mmLbL-INS microspheres every 3 days.

To further investigate the *in vivo* biocompatibility and degradability of the mmLbL-INS microspheres, the sizes of skin protrusion caused by the subcutaneous injection were monitored over time (Figure 5E). The average lump size in the injection sites of rats treated with mmLbL-INS microspheres rapidly decreased in 1 h, as the tissue absorbed moisture into the mmLbL-INS microspheres. In the following days, the average lump size steadily decreased, suggesting that glucose-mediated degradation was substantially triggered (Supplementary Figure S6). No significant skin protrusion can be found after 10 days maybe due to the clearance of phagocytosis (Figure 5F).

### Biosafety of mmLbL-INS microspheres

**FIGURE 6:**
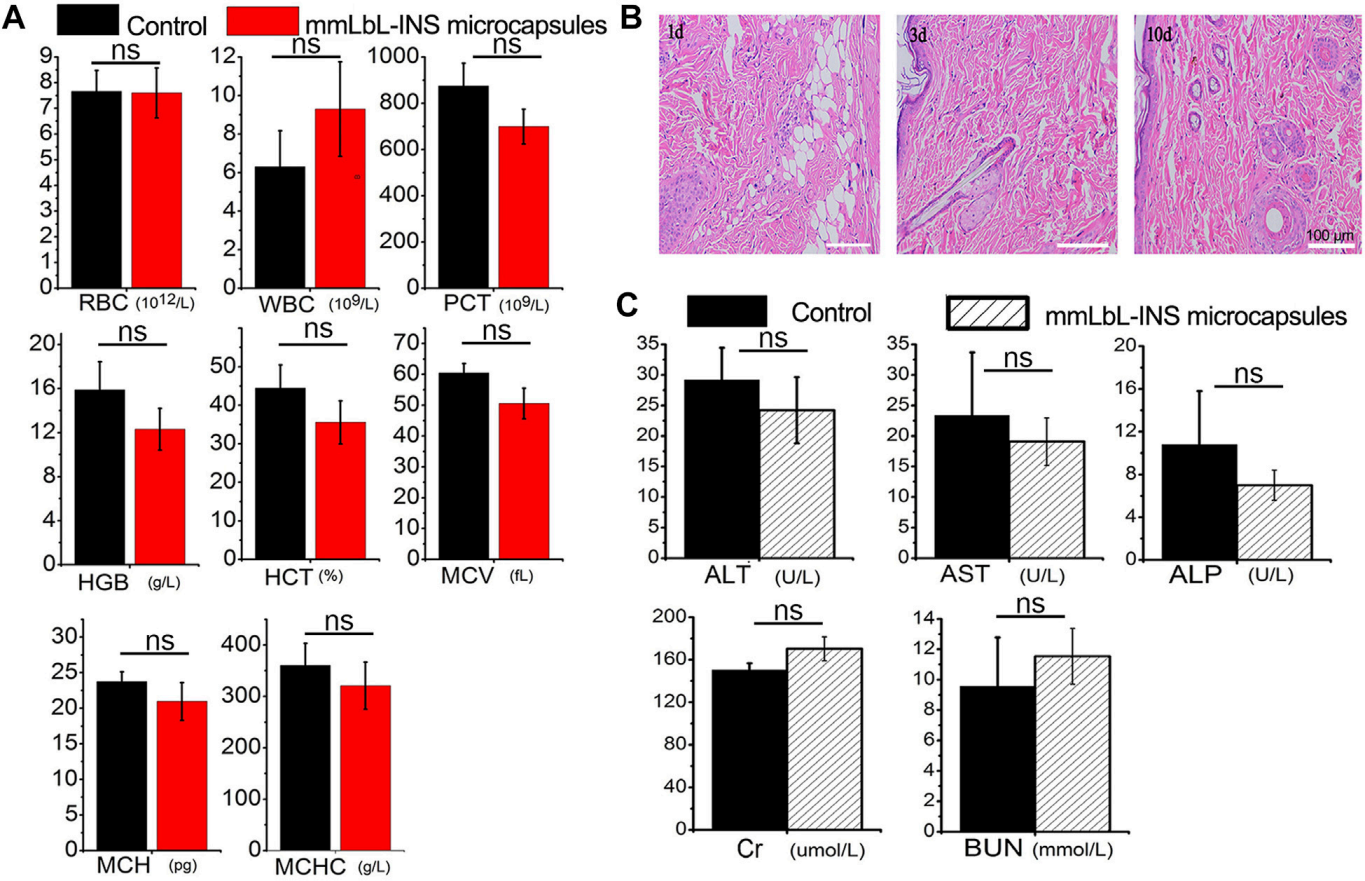
Biosafety of the mmLbL-INS microspheres. **(A)** Hematological parameters after administration of mmLbL-INS microcapsules for 3 days. Red blood cell (RBC), white blood cell (WBC), platelet count (PCT), hemoglobin (HGB), hematocrit (HCT), mean corpuscular volume (MCV), mean corpuscular hemoglobin (MCH), and mean corpuscular hemoglobin concentration (MCHC) were chosen. **(B)** H&E staining of the injected set. Bar = 100 μm. **(C)** Results of liver function markers, including alkaline phosphatase (ALP), alanine aminotransferase (ALT), and aspartate aminotransferase (AST), the kidney function marker urea nitrogen (BUN), and creatinine (Cr). Ns = not statistically different. (n = 8).

The mmLbL-INS microspheres showed satisfactory biocompatibility *in vitro* (Supplementary Figure S7). A complete blood analysis was performed after injection for 48 h, all parameters of the treated group were normal (Figure 6A). No inflammation was found around the injection site and no pathologic changes were noted through hematoxylin and eosin staining (Figure 6B). The liver function markers and the kidney function marker were normal (Figure 6C), suggesting no injury in rats after treatment with the mmLbL-INS microspheres.

## Discussion

Due to the short half-life of INS *in vivo*, directly providing exogenous insulin may lead to multiple injections with careful dosing (Ling and Chen, 2013). Such widely applied treatment can not realize the on-demand release of insulin, leading to the poor patient compliance, and sometimes hypoglycemia, which probably results in the potential risk of brain damage or death (Alloubani et al., 2018; Kawada, 2018). Developing a “closed-loop” system capable of glucose-activated INS control release behavior was of great significance. In this study, the mmLbL-INS microspheres were stable in physiologically relevant environments. Once the blood sugar level increased, GOx converted a dramatic change in the environmental glucose level to a pH stimulus, and the outer anionic polymer was converted readily back to a cationic polymer through amide hydrolysis. The glucose-activated changes led to changes in the nature of ionic interactions in the multilayers and promoted film disruption. The INS release behavior was accomplished through premixed multiple LbL-INS microspheres with different retention times of INS release.

Various glucose-responsive controlled INS release strategies have been approved to maintain normoglycemia for diabetic patients (Chen et al., 2011; Paez et al., 2013; Li et al., 2015; Llanos et al., 2015; Abebe et al., 2022). An INS reservoir (GOx, CAT, and BSA) and could counter hyperglycemia in diabetic rats over a 1-week period (Chu et al., 2012). A glucose mediated INS delivery (consisting of GOx) presented a long-time diabetes management through an injectable nano-network was reported (Gu et al., 2013). A calcium carbonate templated INS microparticles also was reported for improving the efficiency of INS delivery by inhalation (Schmidt et al., 2014). PAH was chosen as the model polymer in preparation of mesoporous hybrid microcapsules, with high and tunable permeability, good stability and multiple functionalities (Jiafu et al., 2013). Although, great success has been obtained as these INS replacement therapies controlled blood glucose by close-loop release behavior, they showed a slow glucose-responsive rate as the mass transport limitation (Ravaine et al., 2008). There was a lag time of more than 2 h in hyperglycemia, which is not suitable for the practical application (Paez et al., 2013). One of the most critical challenges in diabetes treatment is to design self-regulated INS delivery systems, which could control INS release as soon as possible in response to the change of BGL, just like artificial pancreases. In this study, GOx was assembled with the anionic polymer to construct an ultra-fast glucose-activated anionic polymer. The anionic polymer was regarded stable in physiologically relevant environments and hydrolysis of the charge-shifting anionic polymer under acidic conditions yields a cationic polymer. Based on the LbL assembly of multilayered polyelectrolyte films, with repeat assembly sequences polycation PAH and anionic polymer, we fabricated LbL-INS microspheres with different layers and mixed multiple LbL-INS (mmLbL-INS) microspheres proportionally to control the INS release rate in response to the BGL. The results of the mmLbL-INS microspheres showed that it was glucose-mediated INS release and an enhanced hypoglycemic effect for diabetes care. Once the blood sugar level increased, GOx converted a change in the environmental glucose level to a pH stimulus, and the outer anionic polymer was converted readily back to a cationic polymer through amide hydrolysis upon exposure to acidic environments. The results of *in vitro* INS release suggested that glucose can modulate the mmLbL-INS microspheres in an ultrafast glucose activated INS control release and pulsatile profile. *In vivo* studies also validated that this formulation enhanced the hypoglycemic effect in STZ-induced diabetic rats within 2 h of subcutaneous administration. Clinically, patients start on a total daily dose of about 0.5 U/day of INS per kg, and about 30 U of INS is needed daily (adult body weight ∼60 kg). From the results in Figure 2, the INS concentration in mmLbL-INS microspheres could achieve (0.98 mg/ml, roughly corresponding to 28 U/mL). That is to say, a single injection of this mmLbL-INS microspheres into the diabetes patient can maintain the BGL in the normal range about 1 day. To improve the feasibility of clinical application, we could increase the INS content in mmLbL-INS microspheres or increase the injection volume.

In conclusion, this study presented a new glucose-activated mmLbL-INS microsphere INS delivery system as a promising therapeutic strategy in the sustained treatment of hyperglycemia. The LBL assembly of the polyelectrolyte could increase the stability of CaCO_3_-INS, and glucose responsiveness was realized through the charge-shifting effort in aqueous environments by using GOx, which thus resulted in an increased INS release rate, without compromising the BGL response time. *In vivo* results showed that mmLbL-INS microspheres could overcome hyperglycemia within 2 h and maintain the baseline glucose level up to 2 days. This glucose-activatable LbL microspheres system can be a promising alternative in diabetes therapy and serve as a powerful tool for constructing a precisely controlled INS release system.

## Data Availability

The original contributions presented in the study are included in the article/Supplementary Material, further inquiries can be directed to the corresponding author.
