# Supplementary material for: Glucose-activatable insulin delivery with charge-conversional polyelectrolyte multilayers for diabetes care
Source: Front Bioeng Biotechnol. 2022 Sep 29;10:996763. doi: 10.3389/fbioe.2022.996763 (PMC9557070; doi:10.3389/fbioe.2022.996763)
Supplement: Supplementary file 1 [file DataSheet1.docx]

Supplementary Material

Glucose-Activatable Insulin Delivery with Charge-Conversional Polyelectrolyte Multilayers for Diabetes Care

**Yanguang Yang^1^,** **Xiangqian Wang^1^, Xiaopeng Yuan^1^, Qiwei Zhu^1^, Shusen Chen^1^, Donglin Xia^2^***

^1^ Department of Radiotherapy, Nantong Tumor Hospital, Tumor Hospital Affiliated to Nantong University, Nantong, Jiangsu 226361, China

^2^ School of Public Health, Nantong University, Nantong, Jiangsu 226019, China

*** Correspondence:**Corresponding Author [xiadonglin@ntu.edu.cn](mailto:xiadonglin@ntu.edu.cn) (D.L Xia)

Keywords:

Glucose-activity, Diabetes, Insulin, Charge shifting, Layer-by-layer

## Supplementary Figures


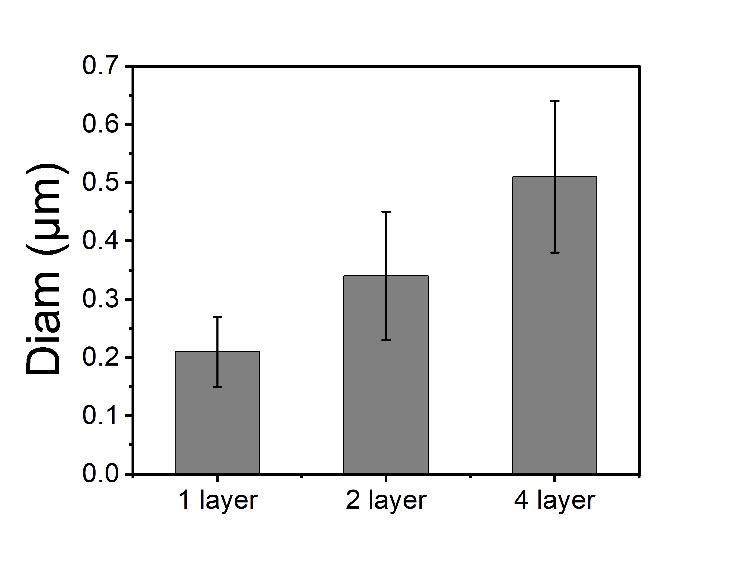


**Supplementary Figure 1.** The change of particle sizes as the polyelectrolyte films increased during the preparation of LbL-INS microspheres.


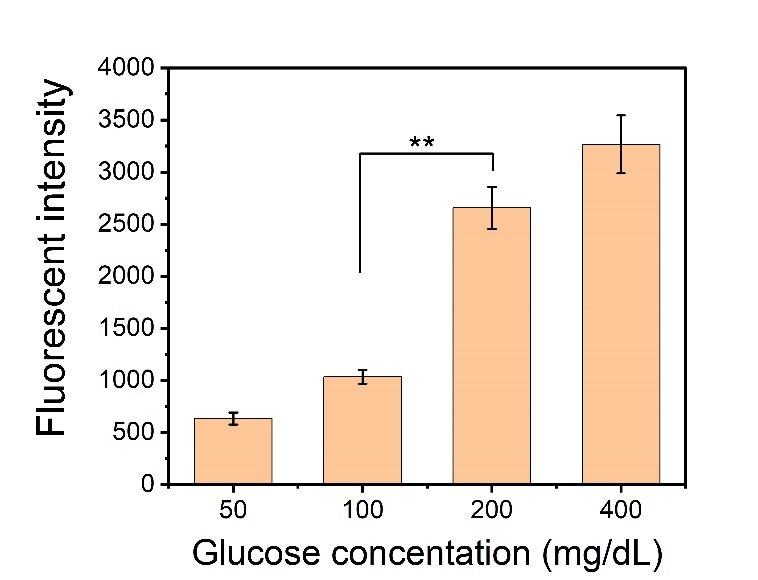


**Supplementary Figure 2.** The fluorescence intensity changes of mmLbL-INS microcapsules that released INS into the medium after being immersed in different glucose concentrations.


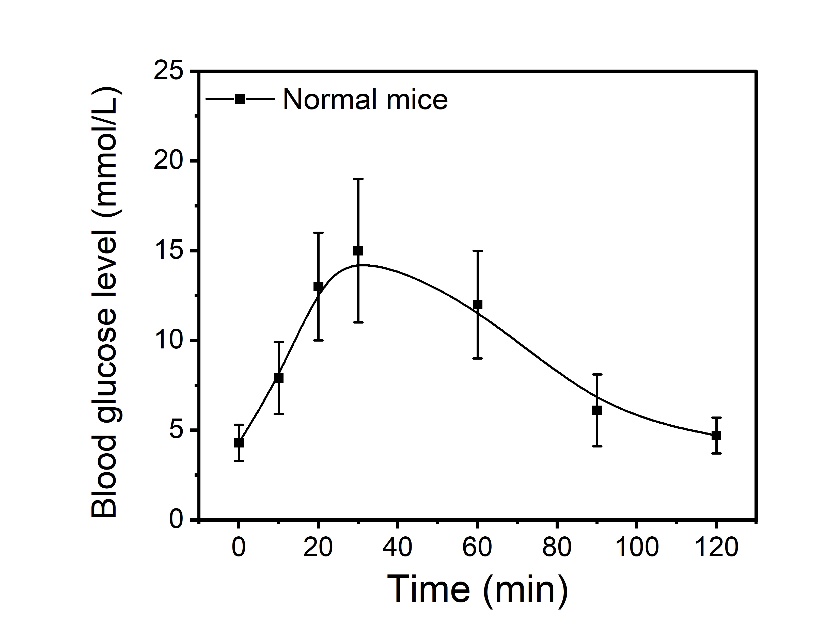


**Supplementary Figure 3.** The curve of normal mice during the OGTT.


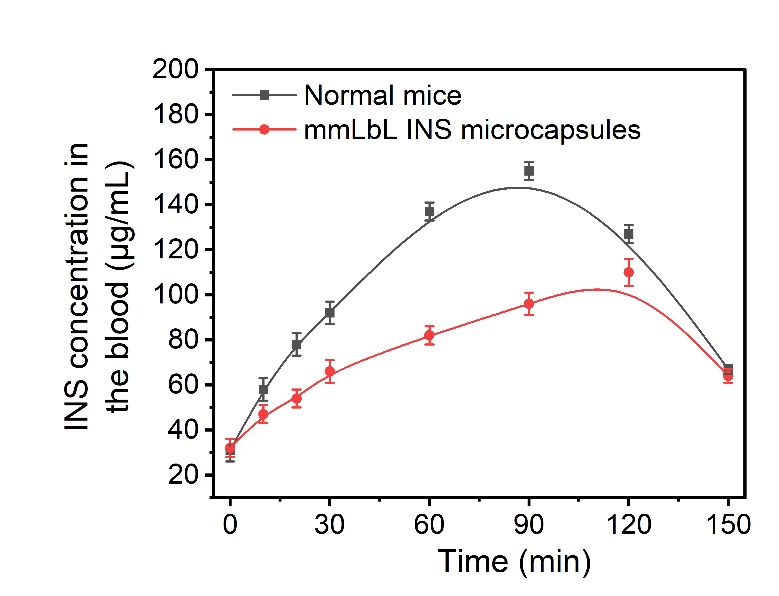


**Supplementary Figure 4.** The change curves of blood INS concentration in normal mice and diabetic rats treated with mmLbL-INS microcapsules.


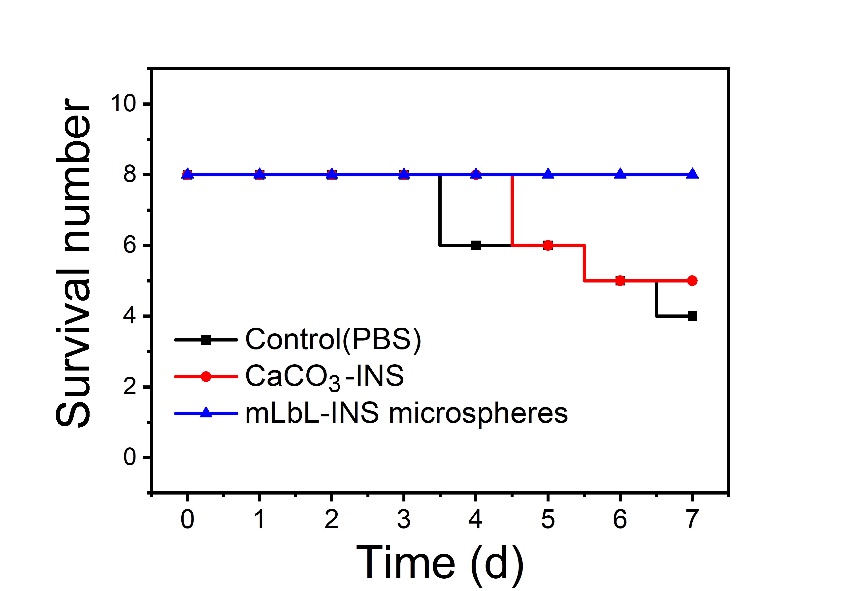


**Supplementary Figure 5.** The change of survival number during the long-term test. The diabetic rats in the mmLbL-INS microspheres treatment group showed no mortality.


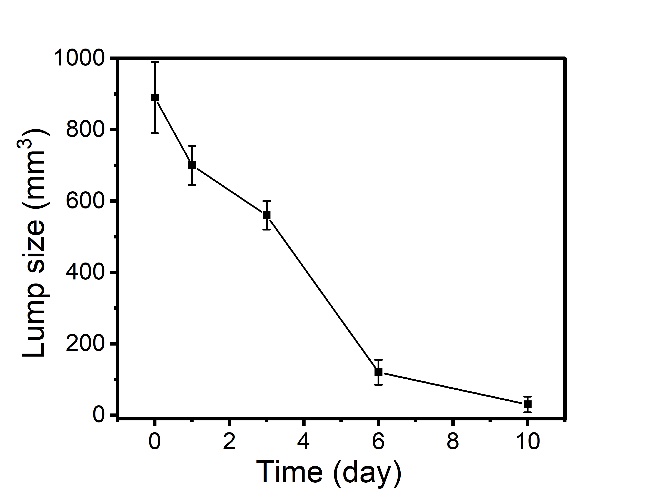


**Supplementary Figure 6.** Changes of lump sizes in the injection sites of diabetic rats treated with 1 mL mmLbL-INS microspheres over time.


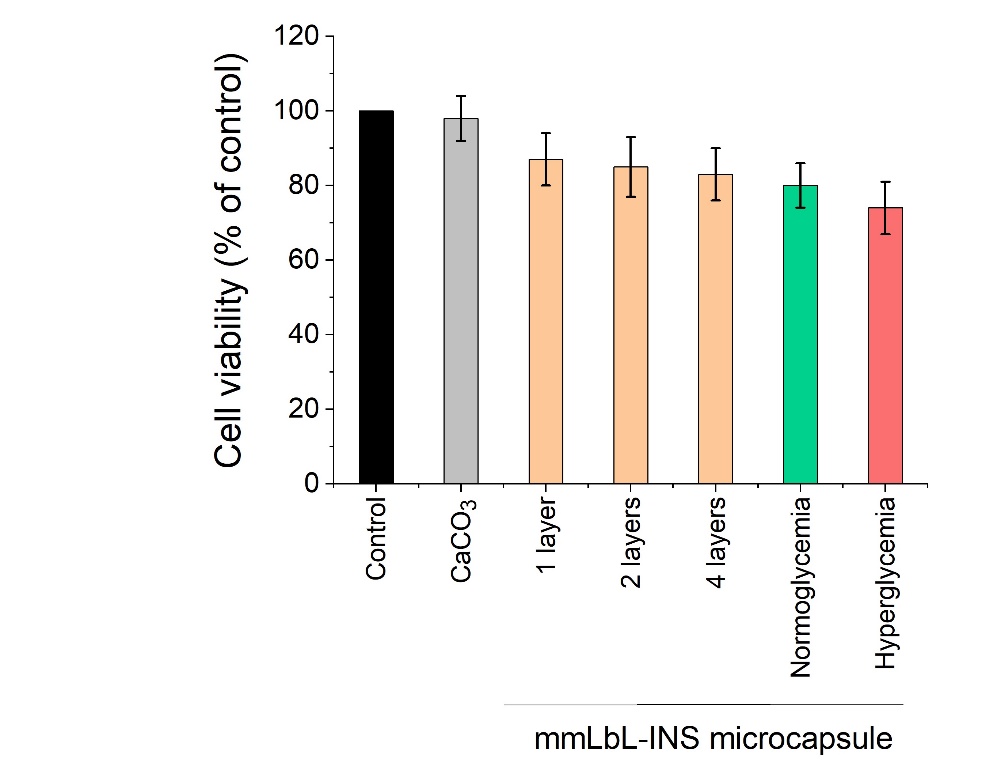


**Supplementary Figure 7.** The biocompatibility of mmLbL-INS microspheres.
